# A Single Belief-Changing Psychedelic Experience Is Associated With Increased Attribution of Consciousness to Living and Non-living Entities

**DOI:** 10.3389/fpsyg.2022.852248

**Published:** 2022-03-28

**Authors:** Sandeep M. Nayak, Roland R. Griffiths

**Affiliations:** ^1^Johns Hopkins Center for Psychedelic and Consciousness Research, Baltimore, MD, United States; ^2^Department of Neuroscience, Johns Hopkins University School of Medicine, Baltimore, MD, United States

**Keywords:** psychedelics, beliefs, 5-HT2A receptor agonist, consciousness, mind perception

## Abstract

**Introduction:**

Although the topic of consciousness is both mysterious and controversial, psychedelic drugs are popularly believed to provide unique insights into the nature of consciousness despite a lack of empirical evidence.

**Methods:**

This study addresses the question of whether psychedelics change the attribution of consciousness to a range of living and non-living entities. A survey was conducted in 1,606 respondents who endorsed a belief changing psychedelic experience.

**Results:**

Participants rated their attributions of consciousness to a range of living and non-living entities before and after their psychedelic experience. Superstitious beliefs and belief in freewill were also assessed. From before the experience to after, there were large increases in attribution of consciousness to various entities including non-human primates (63–83%), quadrupeds (59–79%), insects (33–57%), fungi (21–56%), plants (26–61%), inanimate natural objects (8–26%), and inanimate manmade objects (3–15%). Higher ratings of mystical experience were associated with greater increases in the attribution of consciousness. Moreover, the increased attributions of consciousness did not decrease in those who completed the survey years after the psychedelic experience. In contrast to attributions of consciousness, beliefs in freewill and superstitions did not change. Notably, all findings were similar when restricted to individuals reporting on their first psychedelic experience.

**Discussion:**

This study demonstrates that, among people who reported belief-changing psychedelic experiences, attribution of consciousness to various entities increases. Future prospective psychedelic drug administration studies that control for expectancies are needed.

## Introduction

The definition and nature of consciousness and the closely related problem of other minds, have been debated by scientists and philosophers for centuries, with the topic remaining both mysterious and controversial. Phenomenal consciousness refers to the basic mental state of subjective experience or “what it is like” to be the experiencing entity (Nagel, 1974), while the problem of other minds asks how can we know that anyone or anything other than ourselves has a mind (Avramides, 2020). Although considerable research progress has been made in the field of mind perception, few studies have addressed the causes of mind perception (Gray et al., 2007; Waytz et al., 2010).

Psychedelic substances produce unusual and compelling changes in conscious experience which have prompted some to propose that psychedelics may provide unique insights into the nature of consciousness itself (Yaden et al., 2021). Although it is unclear why psychedelics are closely linked to ideas about consciousness, it is possible that something about the subjective experiences of psychedelics themselves that shape beliefs about consciousness. Indeed, several studies indicate that psychedelic experiences are associated with a range of belief changes including about the fundamental conception of reality itself (Griffiths et al., 2019; Kettner et al., 2019; Davis et al., 2020). A recent prospective study of ceremonial psychedelic use found changes in various metaphysical beliefs, including increased agreement with panpsychism, the notion that that mind or consciousness is a fundamental quality of all things (Timmermann et al., 2021). The present study was undertaken to directly addressed the relationship between psychedelics and beliefs about consciousness, with the results suggesting that a single psychedelic experience increases the attribution of consciousness to a range of living and non-living entities.

## Materials and Methods

### Procedure

In this study an online survey was conducted in individuals who endorsed having a belief-changing psychedelic experience, were at least 18 years of age and were able to read, write, and speak fluent English. Participants answered questions based on a single experience with one several classic psychedelic substances (e.g., psilocybin mushrooms, LSD, ayahuasca). Participants responded to survey items based on the psychedelic experience “that you feel led to the greatest belief change.”

### Participant Recruitment

Participants were recruited from internet postings on social media, relevant websites (e.g., Erowid), and email invitations. Participants were invited to complete an anonymous internet survey of individuals who reported having taken a psychedelic substance that had resulted in a psychedelic experience that led to changes in their beliefs. The belief changes were intentionally described vaguely, specifically participants were invited to take the survey if “You have had changes in your beliefs that you attribute to a psychedelic experience.” Participants were informed that their participation was anonymous, and that they could leave the survey at any time. On the survey landing page, subjects were provided with information about the study and a consent document. The Institutional Review Board of the Johns Hopkins University School of Medicine approved all study procedures (IRB00256742).

### Survey Administration

The survey was designed to take roughly 50 min and to be completed in one sitting. The survey was hosted on Qualtrics.com, a widely used website with suitable security and privacy features for research. There was no compensation.

The survey included questions about demographics, psychedelic use, personality, and scientific knowledge and attitudes. Most of the survey questions focused on belief changes attributed to a single psychedelic experience. Data presented in the current report are only a portion of the larger set which will be reported separately.

#### Details of the Psychedelic Belief-Changing Experience

Similar to prior surveys from our group (Griffiths et al., 2019; Davis et al., 2020), participants were asked to answer questions based on a single reference psychedelic experience. In this case, participants were invited to answer questions based on the psychedelic experience “that you feel led to the greatest belief change.” Participants were asked about which psychedelic they took (psilocybin mushrooms, LSD (Acid), ayahuasca, N,N-DMT (other than ayahuasca), 5-MeO-DMT, mescaline-containing cacti, or other), an estimated dose, whether and what other psychoactive drugs were taken, their age at the time of the experience and when this took place relative to the time of survey administration.

#### Retrospective Ratings of Qualities of the Experience at the Time of the Experience

Mystical-type experiences: Participants were asked to complete the 30-item Mystical Experience Questionnaire (MEQ) (Barrett et al., 2015) according to their feelings, thoughts, and experiences during the reference psychedelic experience. Previous research has shown that mystical experience scores on the day of psilocybin administration predicts subsequent therapeutic and other desirable outcomes (Griffiths et al., 2006, 2016, 2018). Complete mystical experience was defined *a priori* as 60% maximum score on each of the 4 subscales, and coded as a binary variable (Barrett et al., 2015). Total score was expressed as a percentage of maximum possible score.

#### Psychologically Challenging Experiences

As in previous survey and laboratory studies with psychedelics (e.g., Griffiths et al., 2019; Davis et al., 2020) participants were asked to rate on an 8-point scale “How psychologically challenging was the most psychologically challenging portions of the experience,” from 1 = No more than routine, everyday experiences; 6 = Among the 10 most difficult or challenging experiences of my life; 7 = Among the 5 most difficult or challenging experiences of my life; and 8 = The single most difficult or challenging experience of my life. This measure was used as a covariate for examining the specificity of the effects of MEQ (see section “Statistical Analysis”).

#### Ratings of Enduring Effects of Personal Meaning and Psychological Insight Attributed to the Reference Psychedelic Belief-Changing Experience

Enduring personal meaning and psychological insight: As in previous survey and laboratory studies with psychedelics (e.g., Griffiths et al., 2019; Davis et al., 2020), two questions asked participants to rate the degree of personal meaning and personal psychological insight they attributed to the reference experience based “…on your experience and your subsequent contemplation of the experience” on an 8-point scale similar to that used to assess challenging experiences.

#### Ratings of Beliefs Before and After the Experience

Items assessing possible changes in beliefs or worldview attributed to their reference psychedelic experience were rated with 7-point Likert-type items: Strongly disagree (−3), Disagree (−2), Somewhat disagree (−1), Neither agree nor disagree (0), Somewhat agree (+ 1), Agree (+ 2), Strongly agree (+ 3). Participants rated each item at three time points relative to the reference psychedelic experience: “Before (e.g., a month),” “After (e.g., a month),” and “Now.”

#### Belief in the Capacity of Conscious Awareness of Various Living and Non-living Entities

A series of 9 items focused on beliefs in the capacity various living and non-living entities to have conscious experience (see Table 2 for wording of survey items). These items were assessed in the form of a statement such as “Some (if not all) non-human primates (e.g., chimpanzees) are capable of having conscious experience.” The items were presented in what was assumed to represent a descending likelihood of attribution of conscious experience: self, other human beings, non-human primates, quadrupeds, insects, fungi, plants, and inanimate objects. An additional statement “The universe is conscious” was also included.

#### Superstitious Beliefs

Five items from the Revised Paranormal Belief Scale (Tobacyk, 2004) were used to assess superstitious beliefs: (“Black cats can bring bad luck,” “If you break a mirror, you will have bad luck,” and “The number 13 is unlucky”) as well as beliefs that were judged *a priori* to be improbable: “The abominable snowman exists” and “The Loch Ness monster of Scotland exists”

#### Freewill

Belief in freewill was assessed with the question “People have free will; that is, they have the ability to choose between alternative actions.”

### Statistical Analysis

All analyses were performed in R version 4.0.2 (R Core Team, 2021).

Paired *t*-tests and Cohen’s d were used to assess differences in belief agreement ratings between successive timepoints (i.e., Before to After and After to Now). Because large sample sizes can detect statistically significant differences at trivially small effect magnitudes, comparisons between time-points were designated as meaningfully different in the present analysis if they were statistically different (*p* < 1 × 10^–5^) and had at least a small effect size (Cohen’s *d* ≥ 0.2).

For comparison, data on belief agreement was also expressed as the percentage of participants rating any agreement with the belief statement [i.e., Slightly agree (+ 1) to Strongly agree (+ 3)] at each time point. McNemar’s tests were conducted to analyze differences in the proportion of participants endorsing some agreement between successive timepoints (i.e., Before to After and After to Now). Because large sample sizes can detect statistically significant differences at trivially small effect magnitudes, comparisons between time-points were designated as meaningfully different in the present analysis if they were statistically different (*p* < 1 × 10^–5^) and differed by least 10%.

In order to examine the effect of mystical-type experience (MEQ scores) on belief changes, participants were divided into a low and high MEQ groups, with the high group consisting of scores greater than or equal to median MEQ score. Belief agreement ratings of on either side of the median and were compared by paired *t*-tests and Cohen’s d in their differences in belief agreement ratings between timepoints Before to After. In addition, differences between low and high MEQ groups were compared at timepoint “After” by *t*-tests and Cohen’s d. As above, differences were designated as meaningfully different if they were statistically different (*p* < 1 × 10^–5^) and had at least a small effect size (Cohen’s *d* ≥ 0.2).

To further examine the mystical-type experience on belief agreement, linear mixed models with MEQ (as a continuous variable) and Time (Before and After) as factors examined the effect on belief agreement as a continuous variable from −3 (Strongly Disagree) to + 3 (Strongly Agree). Statistical significance was calculated using the ANOVA function of the R package car (Fox and Weisberg, 2019). This analysis controlled for age, age at time of experience, sex, race, whether the experience was the first psychedelic experience, whether this psychedelic experience occurred in the past year, number of lifetime psychedelic uses, which psychedelic was taken, as well as rating of psychological challenge during the experience. This analysis also permitted examination of whether past year psychedelic experience, or first psychedelic experience significantly affected degree of attribution of consciousness.

## Results

### Survey Completion

Respondents were recruited from August 2020 to January 2021. Of the 11,281 individuals who arrived at the survey landing page, 4,997 indicated consent to participate. Subjects were then sequentially excluded as follows: 170 indicated lack of English fluency, 264 indicated they had not had a belief-changing psychedelic experience, 38 failed one or more attention checks, 76 indicated using a drug other than psilocybin mushrooms, LSD (Acid), ayahuasca, N,N-DMT (other than ayahuasca), 5-MeO-DMT, or mescaline-containing cacti, 633 indicated use of multiple psychoactive drugs, and 11 indicated that their age at the time of the reference psychedelic experience was younger than their age at first psychedelic use, and 2,086 did not complete the survey. Qualitative responses in the remaining responses were manually reviewed and a final 113 responses were removed for appearing to refer to multiple psychedelic experiences. The final sample consisted of 1,606 respondents.

### Respondent Characteristics

The final participant population (*N* = 1,606) had mean (SD) age of 35.1 (13.3) years and were predominately white (89%), male (67%), and from the United States (69%). They reported a mean (SD) of 20.9 (25.7) lifetime uses of psychedelics (Table 1). Their reference belief-changing psychedelic experience occurred a mean (SD) of 8.2 (12.6) years before the survey, with 25% of participants indicating the experience occurred in the past 12 months and 44% indicating their reference experience was their first such belief-changing psychedelic experience. The classic psychedelics used at time of reference experience were most commonly psilocybin mushrooms (49%) and LSD (33%).

**TABLE 1:** Participant characteristics (*N* = 1,606).

Age at time of survey completion in years (mean, SD)	35.1 (13.3)
Age at time of reference experience in years (mean, SD)^1^	26.9 (10.1)
Years since the reference experience (mean, SD)^1^	8.2 (12.6)
Lifetime number of uses of a classic psychedelic (mean, SD)	20.9 (25.7)
Reference experience was their first belief-changing psychedelic experience (%)^1^	44%
**Psychedelic used at time of reference experience (%)^1^**	
Psilocybin mushrooms	49%
LSD (Acid)	33%
Ayahuasca	7%
N,N-DMT (other than ayahuasca)	6%
5-MeO-DMT	3%
Mescaline (including Peyote and San Pedro cacti)	2%
Male Sex (%)	67%
**Race (%)**	
White	89%
Asian	6%
Black	1%
Native American	2%
Other	9%
Ethnicity (% Hispanic)	11%
**Marital Status (%)**	
Married and living with spouse	29%
Living with partner	21%
Divorced/Separated	8%
Widowed	1%
Never married	41%
**Country (%)**	
United States	69%
Canada	7%
United Kingdom	4%
Australia	4%
Germany	2%
**Education (%)**	
High School/GED or below	10%
Some College, no degree	21%
Trade school/Associates degree	12%
Bachelor’s Degree	32%
Master’s Degree	17%
Advanced professional degree (e.g., Ph.D./MD)	8%
**Annual household Income (%)**	
Under $25,000	21%
25,000–34,999	10%
35,000–49,999	12%
50,000–74,999	14%
75,000–99,999	11%
100,000–124,999	10%
125,000–199,000	0%
200,000–500,000	9%
Over $500,000	2%

*^1^Reference experience indicates the psychedelic experience that produced the greatest belief-change for the survey respondent. The survey was completed on the basis of that experience.*

**TABLE 2:** Percentage of participants endorsing agreement about beliefs in the capacity of conscious awareness in living and non-living entities (*N* = 1,606).

	Percentage Agreeing^1^		
Belief Statement^2^	Before	After	Now
I (the person taking the survey right now) am capable of having conscious experience.	80%	97%**	98%
Other human beings are capable of having conscious experience.	78%	94%*	95%
Some (if not all) non-human primates (e.g., chimpanzees) are capable of having conscious experience.	63%	83%**	85%
Some (if not all) four-legged animals (e.g., cats, dogs) are capable of having conscious experience.	59%	79%**	82%
Some insects (e.g., ants, flies) are capable of having conscious experience.	33%	57%**	60%
Some fungi (e.g., mushrooms) are capable of having conscious experience.	21%	56%***	62%
Plants (e.g., trees, flowers) are capable of having conscious experience.	26%	61%***	64%
Inanimate natural objects (e.g., rocks) are capable of having conscious experience.	8%	26%**	29%
Inanimate man-made objects (e.g., chairs, buildings) are capable of having conscious experience.	3%	15%*	17%
The universe is conscious.	34%	80%***	82%

^1^Data in these columns show the percentage of participants rating any agreement [Slightly agree (+ 1) to Strongly agree (+ 3)] with the belief statement at each time point. Asterisks indicate that the difference from “Before” to “After” met the criteria for designating a meaningful difference (a difference of at least 10% and a statistically significant difference; *p < 1 × 10^–10^; **p < 1 × 10^–50^; ***p < 1 × 10^–100^). None of the differences from “After” to “Now” met these criteria (Supplementary Material).

*^2^Verbatim wording of belief statements.*

### Qualities of the Psychedelic Experience

About 70% of participants rated the experience as being among the five most personally meaningful and psychologically insightful experiences of their lives (Supplementary Table 1). Ratings of the memory of the experience on the MEQ30 were high (mean 74% of maximum possible score, SD = 20). Almost half of respondents (49%) met *a priori* criteria for a complete mystical experience.

### Changes in Attribution of Consciousness

**FIGURE 1:**
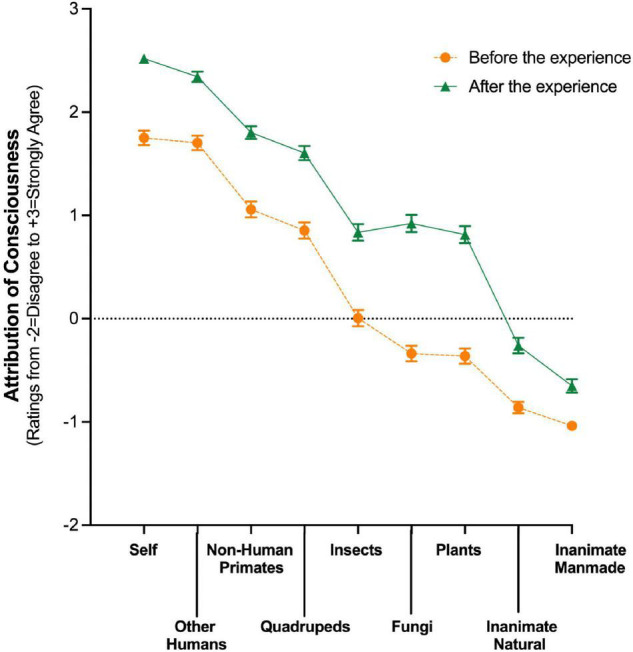
Attribution of consciousness to living and non-living entities before and after a psychedelic belief-changing experience. Y axis: Rating of agreement with the belief that the entity has capacity for conscious awareness from Disagree (–2) to Strongly agree (+3), with Neither agree nor disagree (0) indicated by dotted line. X axes: categories of living and non-living entities. Data are means (±1 SD with yellow circles indicating ratings before and green triangles indicating after the experience. All differences from “Before” to “After” the experience met statistical and effect size criteria for being meaningfully different (Supplementary Material).

Figure 1 shows agreement ratings about belief in the capacity for conscious awareness across each of the 9 living and non-living entities between the timepoints “Before (e.g., a month)” and “After (e.g., a month)” the reference psychedelic experience. Agreement ratings decreased monotonically across the different entities that were sequenced in an order that was assumed to represent a descending likelihood of attribution of conscious experience: self, other human beings, non-human primates, quadrupeds, insects, fungi, plants, and inanimate objects. All differences from “Before” to “After” the experience met our statistical and effect size criteria for being meaningfully different (Figure 1 and Supplementary Table 2). None of the differences from “After” to “Now” (i.e., at the time of the survey) met these criteria. Table 2 shows analogous results expressed as the percentage of participants endorsing any agreement with the belief statement. For example, the percentage of participants agreeing that plants were capable of having a conscious experience increased from 26% “Before” to 61% “After.” Cronbach’s alpha for these items was 0.87 at all three timepoints, indicating adequate internal consistency. Notably, the effects of the psychedelic experience on attribution of consciousness were not affected by whether the psychedelic experience occurred in the past year or whether it was the participant’s first psychedelic experience (data not shown).

**FIGURE 2:**
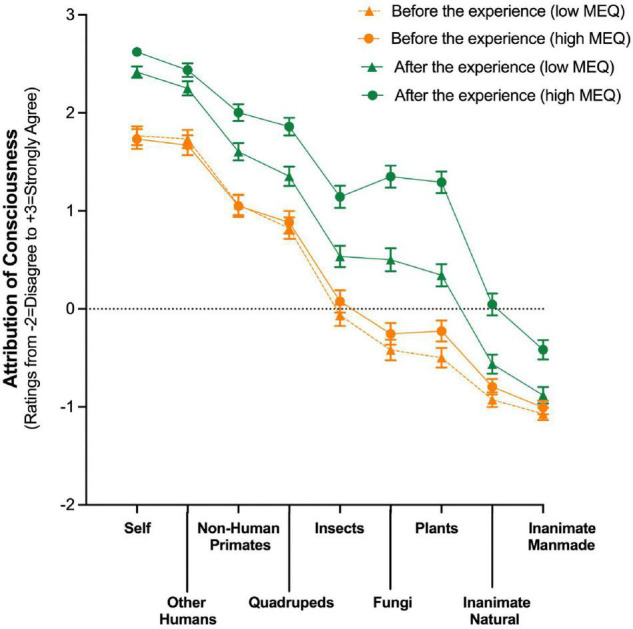
Attribution of consciousness to living and non-living entities after a psychedelic belief-changing experience is greater in those with higher scores on the Mystical Experience Questionnaire (MEQ). Y axis: Rating of agreement with the belief that the entity has capacity for conscious awareness from Disagree (–2) to Strongly agree (+3), with Neither agree nor disagree (0) indicated by dotted line. X axes: categories of living and non-living entities. Data are means (±1 SD); yellow symbols indicate ratings before the experience; green symbols indicate after the experience; triangles and circles indicate the low and high MEQ groups, respectively, with the low group consisting of scores below the median MEQ score. In both the low and high MEQ groups all differences from “Before” to “After” the experience met statistical and effect size criteria for being meaningfully different. Furthermore, with the exception of “Other Humans,” all differences between the low and high MEQ groups met these criteria for being meaningfully different at the “After” timepoint (i.e., the comparison between green triangles and green circles) (Supplementary Material).

Higher scores on the Mystical Experience Questionnaire (MEQ) were associated with greater increases in the attribution of consciousness, as shown in Figure 2 and Supplementary Table 3 when low and high MEQ groups were examined individually. This same relationship was shown when MEQ was examined as a continuous variable after controlling for eight demographic, psychedelic use, and other variables including psychological challenge.

### Changes in Superstitious Beliefs and Freewill

In contrast to changes in beliefs about the capacity of conscious awareness, there were no meaningful changes in ratings of agreement with the five superstitious beliefs items or the belief in freewill item (Table 3 and Supplementary Table 4) and scores on the Mystical Experience Questionnaire did not affect these outcomes (data not shown).

**TABLE 3:** Percentage of participants endorsing agreement about superstitious beliefs and the belief in freewill (*N* = 1,606).

	Percentage Agreeing^1^		
Belief Statement^2^	Before	After	Now
*Superstitious Beliefs*			
Black cats can bring bad luck.	8%	4%	3%
If you break a mirror, you will have bad luck.	10%	5%	4%
The number 13 is unlucky.	7%	3%	2%
The abominable snowman of Tibet exists.	6%	8%	8%
The Loch Ness monster of Scotland exists.	10%	10%	10%
*Belief in Freewill*			
People have free will; that is, they have the ability to choose between alternative actions.	74%	74%	73%

*^1^Data in these columns show the percentage of participants rating any agreement [Slightly agree (+ 1) to Strongly agree (+ 3)] with the belief statement at each time point. None of the differences from “Before” to “After” or from “After” to “Now” met the criteria for designating a meaningful difference (a difference of at least 10% and a statistically significant difference; see section “Statistical Analysis” for rationale).*

*^2^Verbatim wording of belief statements.*

## Discussion

This survey suggests that a single psychedelic experience can increase beliefs that living and non-living entities have the capacity for conscious awareness but does not change beliefs regarding superstitions or freewill. The increases in attribution of consciousness are of large magnitude (show large effect sizes), occur soon after the experience, and are enduring, being unchanged a mean of 8 years after the experience. Strikingly, the high rate of attribution of consciousness to plants (61% of participants after the psychedelic experience) is considerably higher than the 10% or 18% that has been reported in the general population (Arico et al., 2011; Reggia et al., 2015).

The current study showed that the intensity of phenomenological features of the psychedelic experience (assessed with the MEQ) were directly related to the magnitude of increases in the attribution consciousness. This is consistent with the fact that several hallmark features of the mystical experience include a sense of external unity (e.g., MEQ item “Awareness of the life or living presence in all things”) coupled with a noetic quality [e.g., MEQ item (“Certainty of encounter with ultimate reality (in the sense of being able to ‘know’ and ‘see’ what is really real at some time during your experience”) (Barrett and Griffiths, 2018). Thus, a predictable outcome of an experience having an authoritative sense that all things are alive would be increased attribution of consciousness, as shown in the present study.

The finding of the increased broad attribution of consciousness to other entities may be related to the experience of “entity encounters” that are sometimes reported after taking psychedelics and other drugs. In one study (Luke and Kittenis, 2005), 42% of surveyed individuals using psychoactive drugs reported “sensing an intelligence or spirit being in an ingested plant or substance” on the substance, an experience that was commonly reported with several plant or fungi derived drugs including psilocybin mushrooms, ayahuasca, mescaline containing cacti, and also Salvia divinorum. Similarly, two surveys involving psychedelic experiences documented vivid highly meaningful experiences of encountering something that was “conscious (i.e., self-aware)” such as an autonomous entity, ultimate reality, or God (Griffiths et al., 2019; Davis et al., 2020).

Considering attribution of consciousness and the problem of other minds from an evolutionary perspective, the capacity for detecting and attributing agency has self-evident survival value, for instance in the detection of predators. Thus, innate cognitive biases may partially underpin tendencies to attribute mentality to entities without brains. Interestingly, studies suggest that that wide attribution of consciousness may be developmentally normal in children and subsequently suppressed or unlearned (Inagaki and Hatano, 1987; Arico et al., 2011). Perhaps psychedelic mediated neuroplasticity (Ly et al., 2018) facilitates this unlearning by reopening a “critical period” in brain development as has been demonstrated with a single dose of 3,4-methylenedioxymethamphetamine (MDMA) (Nardou et al., 2019). That these experiences may produce compelling experiences that lead to persistent beliefs neither supports nor undermines the epistemic validity of these belief changes.

Limitations of this study include its retrospective design and that it was limited to participants who endorsed a belief change they attributed to a psychedelic experience. It is possible there were demand effects, wherein participants felt compelled to report belief changes along certain directions. However, the lack of change in beliefs about freewill, which is contrary to studies suggesting linkage between agency and attribution of consciousness (Arico et al., 2011; Björnsson and Shepherd, 2020), suggest that the observed changes in attribution of consciousness is not just an artifact of implicit demand characteristics of the survey.

Notably, a recent report comprised of an observational plus controlled psychedelic administration research design showed that that psychedelic administration produced changes in beliefs away from hard physicalism toward dualism that endured 6 months later (Timmermann et al., 2021).

This study demonstrates that, among people who reported belief-changing psychedelic experiences, the attribution of consciousness to various entities increases, which is plausibly related to the widely held belief that psychedelics may provide unique insights into the nature of consciousness. Future prospective psychedelic drug administration studies that include controls for expectancies are needed to better understand the determinants of the robust increases in attribution of consciousness associated with a single psychedelic experience.

## Data Availability Statement

The raw data supporting the conclusions of this article will be made available by the authors, without undue reservation.

## Ethics Statement

The studies involving human participants were reviewed and approved by Institutional Review Board of the Johns Hopkins University School of Medicine. The patients/participants provided their written informed consent to participate in this study.

## Author Contributions

Both authors made substantial contributions to the conception and design of the study, the acquisition and interpretation of the data, drafting of the manuscript, approved the final version of this manuscript, and agreed to be accountable for all aspects of the work.

## Conflict of Interest

RRG is on the board of directors of the Heffter Research Institute. The remaining author declares that the research was conducted in the absence of any commercial or financial relationships that could be construed as a potential conflict of interest.

## Publisher’s Note

All claims expressed in this article are solely those of the authors and do not necessarily represent those of their affiliated organizations, or those of the publisher, the editors and the reviewers. Any product that may be evaluated in this article, or claim that may be made by its manufacturer, is not guaranteed or endorsed by the publisher.
