# Supplementary material for: A Single Belief-Changing Psychedelic Experience Is Associated With Increased Attribution of Consciousness to Living and Non-living Entities
Source: Front Psychol. 2022 Mar 28;13:852248. doi: 10.3389/fpsyg.2022.852248 (PMC8995647; doi:10.3389/fpsyg.2022.852248)
Supplement: Supplementary file 1 [file Table_1.DOCX]

**Supplementary Material**

Nayak, SM and Griffiths RR. A single psychedelic experience increases the attribution of consciousness to living and non-living entities. Frontiers in Psychology.

**This file includes:**

Tables S1 to S4

**Table S1.** Participant ratings of enduring effects attributed to the reference psychedelic belief-changing experience (N=1606)

| *How personally meaningful was your experience?* | | |  |  |
| --- | --- | --- | --- | --- |
| Mean (SD), max score=8 | 6.7 (1.0) | 6. | | |
| % rating top 5 most personally meaningful of lifetime (including single most) | 70% |  | | |
| % rating the single most personally meaningful of lifetime | 19% |  | | |
|  |  |  | | |
| *How personally psychologically insightful was your experience?* |  |  | | |
| Mean (SD), max score=8 | 6.6 (1.5) |  | | |
| % rating top 5 most personally psychologically insightful of lifetime (including single most) | 68% |  | | |
| % rating the single most personally psychologically insightful of lifetime | 26% |  | | |
|  |  |  | | |

**Table S2.** Agreement ratings about beliefs in the capacity of conscious awareness in living and non-living entities (N=1606)

|  | **Agreement Rating** ^1^ | | | **Cohen's d** ^2^ | |
| --- | --- | --- | --- | --- | --- |
|  | **_________________________** | | | **________________** | |
| **Belief Statement** ^3^ | **Before** | **After** | **Now** | **Before to After** | **After to Now** |
| I (the person taking the survey right now) am capable of having conscious experience. | 1.8 (1.4) | 2.5 (0.8) | 2.6 (0.7) | 0.67* | 0.15 |
| Other human beings are capable of having conscious experience. | 1.7 (1.3) | 2.4 (1) | 2.5 (0.9) | 0.53* | 0.11 |
| Some (if not all) non-human primates (e.g. chimpanzees) are capable of having conscious experience. | 1.1 (1.5) | 1.8 (1.2) | 1.9 (1.2) | 0.53* | 0.1 |
| Some (if not all) four-legged animals (e.g. cats, dogs) are capable of having conscious experience. | 0.9 (1.5) | 1.6 (1.3) | 1.8 (1.3) | 0.51* | 0.11 |
| Some insects (e.g. ants, flies) are capable of having conscious experience. | 0 (1.7) | 0.8 (1.6) | 1 (1.6) | 0.53* | 0.07 |
| Some fungi (e.g. mushrooms) are capable of having conscious experience. | -0.6 (1.7) | 0.8 (1.8) | 1 (1.8) | 0.78* | 0.13 |
| Plants (e.g. trees, flowers) are capable of having conscious experience. | -0.5 (1.7) | 0.9 (1.8) | 1.1 (1.8) | 0.8* | 0.09 |
| Inanimate natural objects (e.g. rocks) are capable of having conscious experience. | -1.5 (1.5) | -0.6 (1.9) | -0.5 (1.9) | 0.55* | 0.05 |
| Inanimate man-made objects (e.g. chairs, buildings) are capable of having conscious experience. | -1.9 (1.4) | -1.2 (1.7) | -1.1 (1.8) | 0.43* | 0.05 |
| The universe is conscious. | -0.2 (1.8) | 1.7 (1.5) | 1.8 (1.5) | 1.13* | 0.07 |
| ^1^ Data in these columns show mean (SD) ratings of agreement with each belief statement on a 7-point Likert-type scale from Strongly Disagree (-3) to Strongly Agree (+3) for each of 3 timeframes ("Before (e.g. a month" and "After (e.g. a month)" the reference experience) and at the time of the survey ("Now").  ^2^ Data in these columns show Cohen's d effect size for the comparison from "Before to After" and from "After to Now." Asterisks indicate that criteria for a meaningful difference were fulfilled (i.e. Cohen's d effect size of the difference between time points was ≥ 0.2 AND that the difference was statistically significant). All comparisons meeting the effect size criteria were highly statistically significant, with the p-values ranging from 1.0 x10^-33^ to 1.0 x 10^-196^.  ^3^ Verbatim wording of belief statements. | | | | | |

**Table S3.** Agreement ratings about beliefs in the capacity of conscious awareness in living and non-living entities among participants with low and high Mystical Experience Questionnaire (MEQ) scores (N=1606)

|  | **Low MEQ** ^1^ | | **High MEQ**^1^ | | **Cohen's d** ^2^ | |
| --- | --- | --- | --- | --- | --- | --- |
|  | **_________________** | | **_________________** | | **_____________________** | |
| **Belief Statement**^3^ | **Before** | **After** | **Before** | **After** | **Before to After**  **(Low MEQ)** | **Before to After**  **(High MEQ)** |
| I (the person taking the survey right now) am capable of having conscious experience. | 1.8 (1.4) | 2.4 (0.8) | 1.7 (1.4) | 2.6 (0.7) | 0.56* | 0.79* |
| Other human beings are capable of having conscious experience. | 1.7 (1.4) | 2.2 (1.1) | 1.7 (1.5) | 2.4 (1) | 0.42* | 0.61* |
| Some (if not all) non-human primates (e.g. chimpanzees) are capable of having conscious experience. | 1.1 (1.5) | 1.6 (1.3) | 1.1 (1.6) | 2 (1.2) | 0.38* | 0.66* |
| Some (if not all) four-legged animals (e.g. cats, dogs) are capable of having conscious experience. | 0.8 (1.6) | 1.3 (1.4) | 0.9 (1.6) | 1.9 (1.3) | 0.35* | 0.65* |
| Some insects (e.g. ants, flies) are capable of having conscious experience. | -0.1 (1.6) | 0.5 (1.6) | 0.1 (1.7) | 1.1 (1.6) | 0.38* | 0.64* |
| Some fungi (e.g. mushrooms) are capable of having conscious experience. | -0.5 (1.5) | 0.3 (1.7) | -0.2 (1.5) | 1.3 (1.6) | 0.53* | 0.97* |
| Plants (e.g. trees, flowers) are capable of having conscious experience. | -0.4 (1.5) | 0.5 (1.7) | -0.2 (1.6) | 1.3 (1.6) | 0.57* | 1.00* |
| Inanimate natural objects (e.g. rocks) are capable of having conscious experience. | -0.9 (1.1) | -0.6 (1.4) | -0.8 (1.2) | 0 (1.6) | 0.29* | 0.59* |
| Inanimate man-made objects (e.g. chairs, buildings) are capable of having conscious experience. | -1.1 (0.9) | -0.9 (1.2) | -1 (1) | -0.4 (1.4) | 0.18 | 0.47* |
| The universe is conscious | -0.1 (1.6) | 1.3 (1.6) | 0.1 (1.6) | 2.2 (1.2) | 0.88* | 1.50* |

^1^ Participants were divided into Low and High MEQ groups based on a median split. Data in these columns show mean (SD) ratings of agreement with each belief statement on a 7-point Likert-type scale from Strongly Disagree (-3) to Strongly Agree (+3) for each of 2 timeframes: "Before (e.g. a month)" and "After (e.g. a month)" the reference experience.

^2^ Data in these columns show Cohen's d effect size for the comparison from "Before to After". Asterisks indicate that criteria for a meaningful difference were fulfilled (i.e. Cohen's d effect size of the difference between time points was ≥ 0.2 AND that the difference was statistically significant). All comparisons meeting the effect size criteria were highly statistically significant, with the p-values ranging from 1.0 x10^-8^ to 1.0 x 10^-159^

^3^ Verbatim wording of belief statements.

**Table S4.** Agreement ratings about superstitious beliefs and belief in freewill (N=1606)

|  | **Agreement Rating** ^1^ | | | **Cohen's d** ^2^ | |
| --- | --- | --- | --- | --- | --- |
|  | **_________________________** | | | **_______________** | |
| **Belief Statement** ^3^ | **Before** | **After** | **Now** | **Before to After** | **After to Now** |
| ***Superstitious Beliefs*** |  |  |  |  |  |
| Black cats can bring bad luck. | -2 (1.4) | -2.2 (1.2) | -2.3 (1.1) | -0.13 | -0.07 |
| If you break a mirror, you will have bad luck. | -2 (1.5) | -2.2 (1.3) | -2.2 (1.2) | -0.13 | -0.07 |
| The number 13 is unlucky. | -2.1 (1.4) | -2.3 (1.2) | -2.3 (1.1) | -0.13 | -0.06 |
| The abominable snowman of Tibet exists. | -1.3 (1.5) | -1.2 (1.5) | -1.2 (1.5) | 0.06 | 0.01 |
| The Loch Ness monster of Scotland exists. | -1.3 (1.5) | -1.3 (1.6) | -1.3 (1.6) | 0.03 | -0.01 |
| ***Belief in Freewill*** |  |  |  |  |  |
| People have free will; that is, they have the ability to choose between alternative actions. | 1.3 (1.4) | 1.4 (1.5) | 1.4 (1.6) | 0.02 | 0.01 |
| ^1^ Data in these columns show mean (SD) ratings of agreement with each belief statement on a 7-point Likert-type scale from Strongly Disagree (-3) to Strongly Agree (+3) for each of 3 timeframes ("Before (e.g. a month" and "After (e.g. a month)" the reference experience) and at the time of the survey ("Now").  ^2^ Data in these columns show Cohen's d effect size for the comparison from "Before to After" and from "After to Now." None of these differences met the criterion for designating a meaningful difference (Cohen's d ≥ 0.2 and a statistically significant difference; see Statistics section for rationale).  ^3^ Verbatim wording of belief statement. | | | | | |
